# Circulating vaccine derived polio virus type 2 outbreak and response in Yemen, 2021–2022, a retrospective descriptive analysis

**DOI:** 10.1186/s12879-024-09215-1

**Published:** 2024-03-15

**Authors:** Mutahar Ahmed Al-Qassimi, Mohammed Al Amad, Ahmed Al-Dar, Ehab Al Sakaf, Ahmed Al Hadad, Yahia Ahmed Raja’a

**Affiliations:** 1 National Polio surveillance coordinator, Yemen Ministry of Public Health and Population, Sana’a, Yemen; 2 Department of Community Medicine, Faculty of Medicine and Health Sciences, Sana’a University, Sana’a, Yemen; 3 General Director for Diseases Control and Surveillance, Yemen Ministry of Public Health and Population, Sana’a, Yemen; 4 Faculty of Medicine and Health Sciences, Sana’a university, Sana’a, Yemen; 5 Yemen National Certification of polio eradication Committee chairperson, Sana’a, Yemen

**Keywords:** Circulating Vaccine Derived Polio Virus Type 2, Outbreak, Yemen, 2022

## Abstract

**Background:**

The outbreaks of circulating Vaccine Derived Polio Viruses (cVDPVs) have emerged as a major challenge for the final stage of polio eradication. In Yemen, an explosive outbreak of cVDPV2 was reported from August 2021 to December 2022. This study aims to compare the patterns of cVDPV2 outbreak, response measures taken by health authorities, and impacts in southern and northern governorates.

**Method:**

A retrospective descriptive study of confirmed cases of VDPV2 was performed. The data related to cVDPV2 as well as stool specimens and environmental samples that were shipped to WHO-accredited labs were collected by staff of surveillance. Frequencies and percentages were used to characterize and compare the confirmed cases from the southern and northern governorates. The average delayed time as a difference in days between the date of sample collection and lab confirmation was calculated.

**Results:**

The cVDPV2 was isolated from 227 AFP cases reported from 19/23 Yemeni governorates and from 83% (39/47) of environmental samples with an average of 7 months delayed from sample collection. However, the non-polio AFP (NPAFP) and adequate stool specimen rates in the north were 6.7 and 87% compared to 6.4 and 87% in the south, 86% (195) and 14%(32) out of the total 227 confirmed cases were detected from northern and southern governorates, respectively. The first and second cases of genetically linked isolates experienced paralysis onset on 30 August and 1st September 2021. They respectively were from Taiz and Marib governorates ruled by southern authorities that started vaccination campaigns as a response in February 2022. Thus, in contrast to 2021, the detected cases in 2022 from the total cases detected in the south were lower accounting for 22% (7 of 32) of compared to 79% (155 of 195) of the total cases the north.

**Conclusion:**

A new emerging cVDPV2 was confirmed in Yemen. The result of this study highlighted the impact of vaccination campaigns in containing the cVDPV2 outbreak. Maintaining a high level of immunization coverage and switching to nOPV2 instead of tOPV and mOPV2 in campaigns are recommended and environmental surveillance should be expanded in such a risky country.

**Supplementary Information:**

The online version contains supplementary material available at 10.1186/s12879-024-09215-1.

## Introduction

Despite the progressive reduction in Wiled Polio Virus (WPV) cases globally, where only two countries, Pakistan and Afghanistan, are still endemic with WPV, outbreaks of Vaccine-Derived Polioviruses (VDPVs) have emerged as a major challenge for the final stage of polio eradication efforts.

VDPVs are classified as circulating if there is evidence of community transmission (cVDPV), immunodeficiency-associated if isolated from a person with an immunodeficiency (iVDPV), or ambiguous when there is neither an immune-compromised individual nor evidence of community circulation (aVDPV) [1–3]. 

VDPV is a strain of the poliovirus that has been genetically mutated from the strain contained in the oral polio vaccine (OPV). It is caused by a rare mutation that occurs when the weakened virus in the vaccine is allowed to circulate among under-immunized populations for an extended period of time.

This divergent effect allows the attenuated vaccine virus to regain its virulence and cause paralysis and death, especially in areas with low vaccination coverage.

After the global switch from trivalent OPV (tOPV) (types 1, 2, and 3) to bivalent OPV (bOPV) (types 1, and 3) in April 2016, the number and geographic scope of cVDPV2 outbreaks have extended. From May 2016 to December 2021, about 2298 lab-confirmed cases were reported, distributed across 48 countries, most of them in Africa [4].. The highest number, 1080, was recorded in 2020, followed by 682 cases in 2021. Furthermore, in the same period, the environmental surveillance in those countries detected 1338 positive samples [3, 5].

In Yemen, the first cVDPV2 outbreak was in 2011–2012, when nine cases of cVDPV type 2 were detected by AFP surveillance in six governorates, including; Al Hodeida, Ibb, Hajjah, Amran, Saadah, and Sanaa city [6]. 

In April 2016, as recommended by WHO global scheduling, Yemen withdrew tOPV and shifted to bOPV. The first inactivated poliovirus vaccine (IPV) dose was administered in the country in mid-2015, with one dose administered with the third dose of Penta vaccination, in addition to five bOPV doses in routine vaccination. The last detected VDPV2 was in June 2016, ambiguous type aVDPV2, in a case from Aden governorate [7]. 

In 2020, cVDPV1 outbreak with 31 confirmed cases restricted in Saadah governorate was reported. This outbreak extended to 2021, when three cases were reported from Saadah, the last one in March 2021 [8]. 

Between August 2021 and December 2022, an explosive outbreak of cVDPV2 with 227 confirmed cases was reported [4, 9].. This study aims to compare the patterns of cVDPV2 outbreak, response measures taken by health authorities, and impacts in southern and northern governorates.

## Methods

### Background

**Fig. 1:**
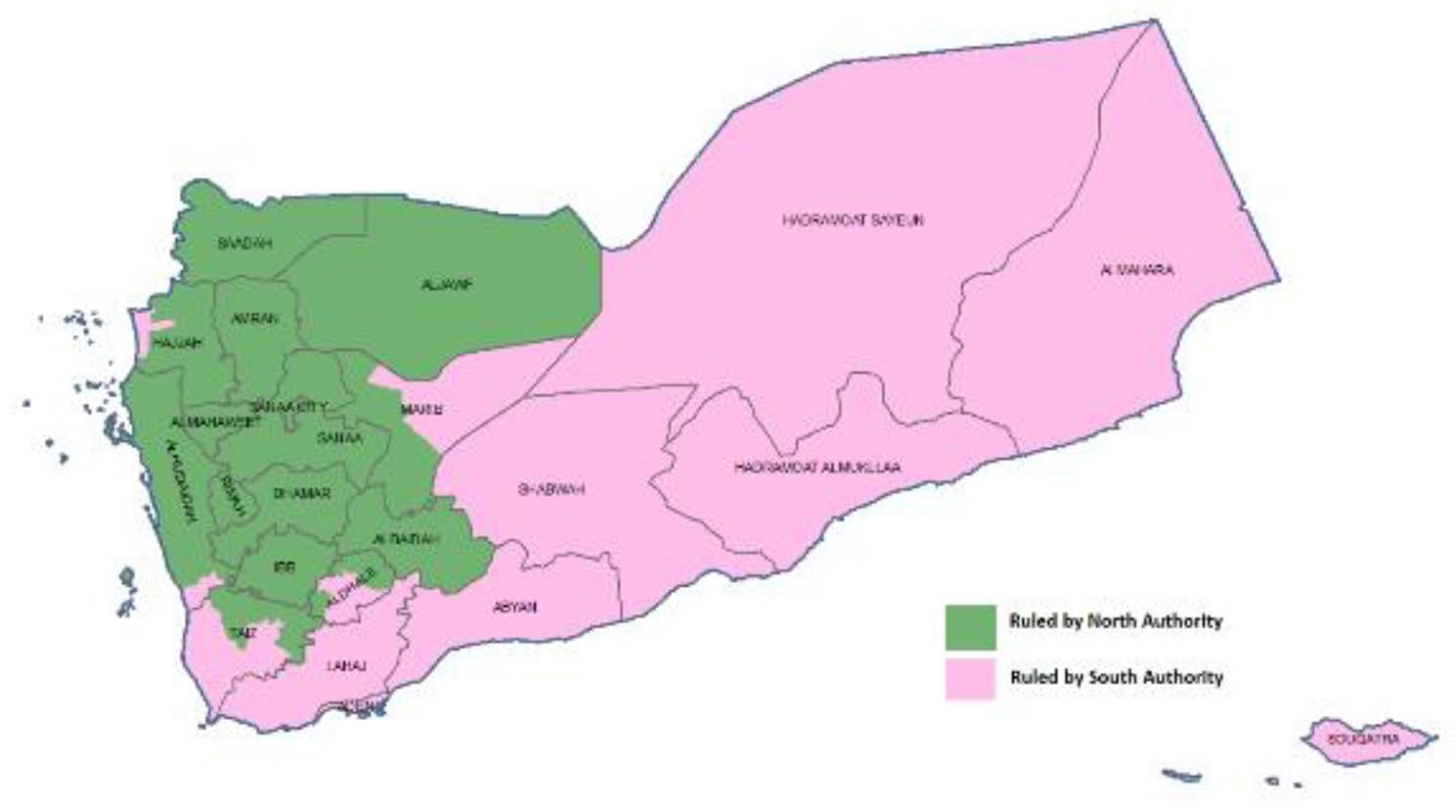
Map of geographical areas controlled by two authorities in Yemen 2022

Yemen is one of the conflicting countries in the Eastern Mediterranean Region. It has experienced the largest humanitarian crisis in the world. Since the start of the war in 2015, the country has been ruled by two authorities, one in the north and another in the south. The bulk of the population, 75% (25,699,313), is in the north, whereas the majority of areas are in the south. Administratively, the country is divided into 333 districts, 217 of which are under the control of the north authority and 116 of which are under the control of the south authority [10]. (Fig. 1)

As a consequence of the war and humanitarian crisis for about 8 years in Yemen, the health system infrastructure is mostly destroyed, routine immunization (RI) coverage is low [11], and significant numbers of vaccine-preventable disease outbreaks such as diphtheria and measles are reported.

### Case ascertainment and definition

In 1998, the Yemeni Ministry of Health, with the support of World Health Organization (WHO), established the Acute Flaccid Paralysis (AFP) surveillance system to detect paralytic poliomyelitis.

Despite the political division in the country, the system is still unified and functioning as a national system for all parts of Yemen. The activities of the AFP surveillance system, as described in the previous study [8], include AFP case detection, reporting, investigation, sample collection, active search, and flow-up assessment of AFP cases after 60 days of paralysis onset. The WHO definition for VDPVs was used; an AFP case with genetic sequences from at least one stool sample shows Sabin-related poliovirus divergence (≥ 6% nucleotides (nt) in genomic part VP1) for PV type 2. Epidemiological information on source patients and isolates was collected by surveillance staff. Epidemiological case investigation for each AFP case is conducted and the data of cases, collected samples, and results are kept at national AFP surveillance in the general directorate for disease control and surveillance.

### Environmental surveillance for poliovirus

The establishment of environmental surveillance in the country was planned to start in 2017, but due to the crisis situation, it started in July 2021. Three governorates, including Sanaa City, Al-Hodeida governorates (located in the north) and Aden governorate (located in the south), were selected and specimens from the main swage stations were started on a monthly basis. The bag-mediated filtration system (BMFS) method, which is a new environmental surveillance tool, was used. Sample volumes of up to 6 L of wastewater using gravity filtration were collected. This method offers higher sensitivity as it samples a large volume; it is able to sample volumes of wastewater 10 to 20 times greater than the two-phase method, and the small filters are easier to ship from remote and challenging environments than liquid samples. The use of preservatives on the filter reduces the need for immediate processing when the filters are received at a reference laboratory [12, 13]. 

The collected virocap filters are sent to the national Center for Public Health Laboratories (CPHL), where preservation agents are added to them and stored in the cold chain until the time of shipment.

### Data management and analysis

The surveillance data of the AFP surveillance program, data of environmental surveillance, reports related to cVDPV2 outbreak and response measures, e.g. data of vaccination campaigns in each region were used. The data included demographic variables for confirmed cases (age, sex, location), date of paralysis onset and vaccination status of confirmed cVDPV2 cases. Date of environmental sample collection and results. Dates of implemented vaccination campaigns, targeted population, places and coverages.

A retrospective descriptive study of all children affected by cVDPV2 during 2021–2022 was performed.

Epi-Info version 7.2 was used for data analysis. A descriptive statistic: frequencies and percentages were used to compare the characteristics of confirmed cases from southern and northern governorates. The average delayed time for lab confirmation as the difference in days between the date of sample collection, shipment, and receiving lab result was calculated.

## Results

### Detection of vaccine-derived polioviruses cases

Referral lab results on 22 November confirmed the isolation of a new emergence of VDPV2 from an AFP case (the index case), reported on 31 of August 2021, from Thubab district, which is located in the part of Taiz governorate that is ruled by southern authority. It was a female aged 108 months (9 years), she had not been vaccinated against polio and experienced onset of paralysis on 30 August 2021. The second isolation, genetically linked to the first one, was from a 26-month-old girl reported from Marib district, Marib governorate. The child had also not been vaccinated against polio and experienced onset of paralysis on 1st September 2021, which indicated a new emergence of cVDPV2 in the country.

Up to December 2022, a total of 227 lab-confirmed cVDPV2 cases were reported from 19/23 Yemeni governorates, including 7% (16/227) reported from 2 governorates (Tazi and Marib), of whom some districts were partially ruled either by north or south authorities, 9% (21/227) reported from 6 governorates ruled by south authority and 84% (190/227) reported from 11 governorates ruled by north authorities. Generally, 86% (195/277) of the confirmed cases were reported from the northern governorates, and 14% (32/277) were reported from the southern governorates.

The first detected case (Index case) in Taiz governorate was followed by four cases reported in September; one case was reported from the same governorate, and three cases were from Marib, Abyan and Aden governorates, which are ruled by south authority. The first confirmed cVDPV2 cases from northern governorates were reported from another part of Taiz governorate that is ruled by north authority (supplementary Table 1).

The confirmed cVDPV2 cases from Northern governorates started in October 2021, (two months of the index case) when the confirmed cases had increased to reach 14 cases (3 cases from the South vs. 11 cases from the north). The detected cases reached the highest peak of the Epi curve in January 2022 with 35 cases (4 from the south vs. 31 from the north).

**Fig. 2:**
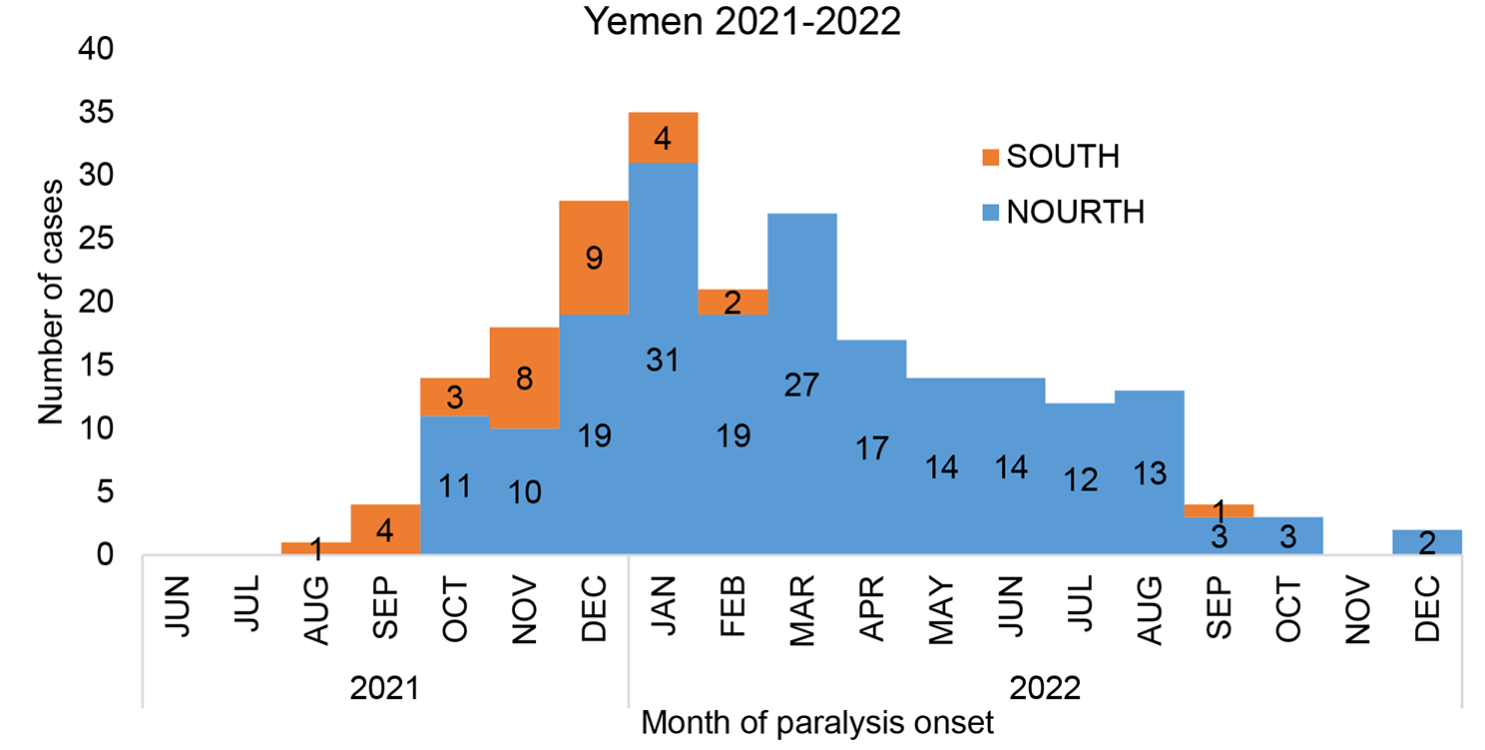
Epi curve of confirmed case by month of paralysis onset, Yemen 2021-2022

By February 2022, the number of detected cases decreased to 21 cases (2 from south vs. 19 from north). In March 2022, the number of reported cases from south reached zero cases while 27 cases were reported from the north (Fig. 2).

Out of the total 227 confirmed cases, 29% (65) were reported in 2021, 91% (207) were below 5 years old, the median age was 18 months, ranging from 3 to 156 months, 63% (142/227) were males and 82% (187) had zero doses of IPV. The confirmed cases from the North compared to the confirmed cases from the South accounting for 21% (40 of 195) and 78% (25 of 32) of the reported cases in 2021 compared to 79% (155 of 195) and 22% (7 of 32) of the reported cases in 2022 from the north and south, respectively. (Table 1)

**Table 1:** Characteristics of reported cVDPV2 cases from north and south Yemen, 2021–2022

	Whole country		North		South	
**Cases of cVDPVs**						
**Characteristics**	**No.**	%	**No.**	%	**No.**	**%**
	227	100%	195	86%	32	14%
**Year**						
2021	65	29%	40	21%	25	78%
2022	162	71%	155	79%	7	22%
**Age group**						
< 5 Ys	207	91%	179	92%	28	88%
> 5 Ys	20	9%	16	8%	4	12%
**Sex**						
Male	142	63%	124	64%	18	56%
Female	85	37%	71	36%	14	44%
**IPV in RI**						
Zero dose	187	82%	158	81%	29	91%
One dose	40	18%	37	19%	3	9%
**Main AFP surveillance indicators**						
**Years**	**NPAFP rate** **6.6**	**%ADEQ** **87.5%**	**NPAFP rate** **6.7**	**%ADEQ** **87%**	**NPAFP rate** **6.4**	**%ADEQ** **88.5%**
2021	5.5	88%	5.6	87%	5.3	89%
2022	7.7	87%	7.8	87%	7.4	86%

NPAFP; non polio acute flaccid paralysis/100,000 population < 15 years, ADEQ; # of AFP cases with 2 stool specimens collected > = 24 h apart and < = 14 days of onset, RI: Routine Immunization, IPV: Inactivated Polio Vaccine

### Close contacts

For each inadequate AFP case, samples from three close contact are collected by AFP surveillance program. Out of the total 1046 samples collected in both years, 6%(65) were lab-confirmed cVDPV2, including 7%(35/ 499) and 5% (30 / 547) of samples collected in 2021 and 2022, respectively. The majority of the confirmed samples 88% (57/65) were from northern governorates.

### Environmental samples

With the help of WHO, 28 ViroCap filters collected from July 2021 to May 2022 have been sent through five shipments to KEMRI in Nairobi, Kenya, whereas 20 ViroCap filters collected from June 2022 to December 2022 have been sent through two shipments to the Pakistan polio lab.

The cVDPV2 was isolated from 83% (39 of 47) of environmental samples and accounted for 93% (13 of 14) and 79% (26 of 33) of the collected samples in 2021 (6 months) and 2022, respectively.

**Fig. 3:**
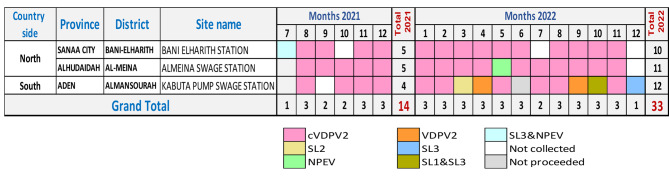
Environmental samples collected with lab results 2021–2022

From the 39 positive cVDPV2 samples, 74% (29/39) were collected from the two stations in the north, and the rest, 26% (10/39) were collected from the collecting site in the south. (Fig. 3)

Due to the challenges of samples transportation in the country and the limited capacity of WHO-accredited polio labs dealing with BMFS samples the average of delayed days between ES collection and lab results were 221 and 210 days (7 months) in 2021 and 2022, respectively. (supplementary Table 2).

### Genetic analysis

The genomic sequences of isolated cVDPV2 virus revealed that they diverged between 9 and 31 VP1 nucleotides (nt) from Sabin like 2 which not genetically linked to any previously sequenced VDPVs.

Two emergence strains of cVDPV2 were reported in this outbreak: the first main cluster, YEM-TAI-1, was isolated from the index case, and another cluster, YEM-SAN-1, was isolated from ES from Bani Elharth sewage station in Sanaa City. Only five AFP cases and one ES were related to the second cluster, and the majority of cases and ES were linked to the first strain.

### Immunization response and impact

As a response, three rounds of tOPV vaccination campaigns were implemented only in the southern area. Two consecutive rounds were implemented on [4, 19–23] February and on [4, 19–23] March 2022, respectively. The two campaigns targeted 2,450,000 children under 10 years old, distributed in 120 districts of 12 governorates located in the southern part, and achieved coverage of 90% and 96%, respectively. The third round was implemented on (27–29) June 2022, targeted 1,192,862 children under 5 years in the same districts, and achieved administration coverage of 104%. (Table 2)

**Table 2:** Polio vaccination campaigns in response to cVDPV2 outbreak, southern Yemen, 2022

Round	Date	Vaccine	location	Target age	Target population	Vaccine coverage
First	19–24 Feb-2022	tOPV	120 districts in 12* Provinces Southern Yemen	< 10 years	2,456,114	90
2nd	19–24 Mar-2022	tOPV		< 10 years	2,357,414	96
3d	27–29 Jun-2022	tOPV		< 5 years	1,192,862	104

tOPV: trivalent Oral polio Vaccine

*116 and 4 districts implemented partially as these districts divided between south and north

Although there is a delayed implementation of vaccination campaigns in the southern governorates, there were dramatic drops in positive cases after the February round, and no cVDPV2 cases were reported from the southern governorates until September 2022, when one case was reported. This case lives in border areas between the South and the North. The epidemiological investigation showed zero doses of polio vaccines, and the case had not been vaccinated at any of the three rounds implemented in the South.

**Fig. 4:**
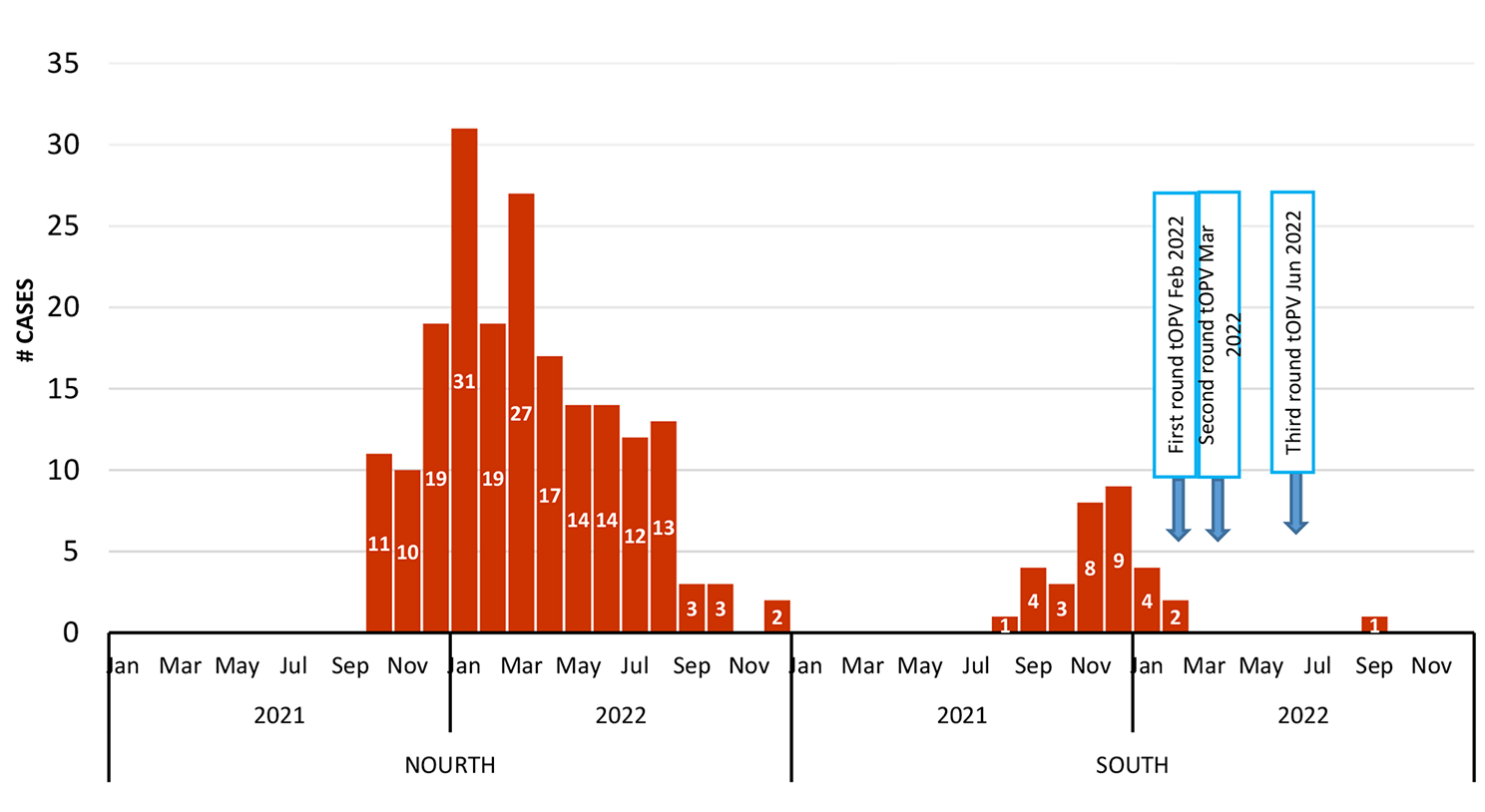
Time distribution of cofirmed CVDPV2 cases and implimented vaccination campaigns, Northern and Souther Yemen 2021-2022

In contrast, the number of confirmed positive cases in the northern governorates continued to fluctuate between increases and decreases, reaching 2 cases in December 2022. (Fig. 4)

## Discussion

This study describes the cVDPV2 outbreak in one of the Eastern Mediterranean conflict countries, such as Yemen, where the confirmed cVDPV2 cases accounted for one-third of the global reported cases in 2022 [14]. The study provides an example of conflict countries where the outbreak is exacerbated by many contributors, such as health system collapse, incapacity for testing and difficulties in samples transportation [11, 15]. 

The finding indicated a new emergence of VDPV2 outbreak in Yemen, which was confirmed in 2021. Yemen switched from tOPV to bOPV in April 2016 and the last confirmed case of VDPV2 was ambiguous type aVDPV2 in June 2016. The new emergence of cVDPV2 after nearly 5.6 years of the last confirmed case might be due to a vaccine virus (most often mOPV2 ) that probably entered the country through population movement from Horn of Africa countries (HOA), where since year 2018 large-scale campaigns with mOPV2 in response to cVDPV2 have been implemented, and from January to December 2018 about 834,665 migration movements from HOA to Yemen were reported [16–20]. The huge influx of migrants from Ethiopia and less extended from Somalia to Yemen can carry a risk of polio viruses transportation and might be the main contributor [21]. 

Our result showed the outbreak of the new emergence VDPV2 started in the southern part of Yemen, particularly in Thubab district and within a month spread to three governorates in the south and within two months with a higher number of cases to another part of Taiz governorate and four governorates ruled by northern authorities. This finding could be attributed to the coastal location of Thubab district, which could be used as a transit point for Ethiopian migrants who then either move to the southern or northern governorates. The higher number of cases in the northern governorates could be attributed to the higher population of under-vaccinated children and the prolonged circulation of the virus in the northern governorates compared to the southern governorates.

The result indicated that by the end of February 2022, i.e., six months after the first confirmed case, cVDPV2 cases were reported from 19 governorates. The emergence and circulation of cVDPV2 within this short period might be explained by the decline of intestinal mucosal immunity levels against serotype 2 poliovirus after the switch from tOPV to bOPV in 2016.

Similar to the result of the previous study, which revealed a delay in lab confirmation [8] the result of this study showed a delay of 86 days between the detection of the second linked case and the confirmed lab result and 176 days between the lab result of the second isolate and the implementation of the first round of vaccination campaigns in the south.

The findings in this study showed that, in contrast to the north governorates, the majority of confirmed cases were detected in 2021 and no cases were detected after February 2022, while the majority of reported cases from the north were detected after February 2022. The difference is attributed to the implementation of two large-scale house to house vaccination campaigns in south governorates. This result indicated evidence for the effectiveness of the house-to-house vaccination strategy campaigns to stop the circulation of cVDPV2.

While most of the countries affected by cVDPV2 outbreak used mOPV2 and recently nOPV2, the country chose tOPV3 in response because the country suffered from cVDPV1 outbreak in 2020–2021.

nOPV2 is a more genetically stable vaccine than Sabin OPV2, which was created to reduce the possibility of reversion to neurovirulence.

The use of nOPV2 in outbreak response to cVDPV2 is required for a timely response, high quality, and sufficient reach [4, 22]. 

Our result showed only four governorates haven’t reported cVDPV2 cases: one in the north and three in the south. Ramah governorate in the north, which is a hard to reach, mountainous area with difficult terrain and is not easy to access, has lower population movement compared with other northern governorates. The three governorates in the south, including Soquatra, which is an island, Al-Mahra, which is an eccentric desert governorate and Hadrmout Al-Muklaa have low population movements. Furthermore, the implementation of three rounds of house-to-house campaigns in the southern governorates may contribute to stopping the circulation of virus there.

The majority of lab-confirmed cVDPV2 from both north and south governorates had zero doses of IPV, this indicates insufficient routine immunization coverage.

The result also showed 18% of children who received one dose of IPV were affected. This might be due to the fact that one dose of IPV gives only 33%, 41%, and 47% protection for serotypes 1, 2, and 3, respectively [15]. This finding has shown a big challenge in the immunity profile of children against type 2, thus IPV routine vaccination should be administered in three doses to achieve full protection of children all around the world [23]. 

Our result showed the sensitivity of ES, even with the prolonged delay of testing (221 days) and spread of the virus in most governorates. The isolation of the virus from all ES since August 2021 up to November 2022 revealed a sensitive ES and an explosive outbreak, so the delay in ES testing should be addressed and managed to improve the timeliness of response procedures.

Our findings showed that the cVDPV2 virus was isolated from stool samples and ES in Yemen, which is consistent with the findings in Egypt and Djibouti [4]. 

Several countries have reported that polioviruses (VDPVs) circulating in the population have also been found in environmental sewage. For instance, in Sudan in September 2020, the virus was detected both in the AFP cases and sewage in Egypt [24]. Similarly, the cVDPV2 virus responsible for a large outbreak in Afghanistan has been identified in sewage in two districts of Iran and in two cases of acute flaccid paralysis (AFP) in Tajikistan [17, 24]. These findings suggest that environmental surveillance can be an effective tool for identifying circulating VDPVs outbreaks and closing gaps in detecting polioviruses during the endgame of polio eradication.

There are some limitations to this study. It is based on secondary data collected by surveillance staff. It did not address the immediate response to the confirmed cases as it was late due to delayed lab confirmation and limited due to the security situation. Although there are many limitations, the study provided information on the impact of providing a large-scale campaign to stop such an outbreak and provides an example of the effectiveness of large-scale vaccination as containment measures for cVDPV2 in conflict areas.

## Conclusion

After nearly 5.6 years since the last confirmed case of ambiguous type aVDPV2, a new emerging VDPV2 outbreak was confirmed, and within six months of the first confirmed case. Many factors have been contributed to the spread of cVDPV2 to the majority (19/23) of governorates: the low population immunity as an impact of insecurity on immunization coverage, the delay of response as a consequence of lab confirmation delay. However, six-month delay in implementing vaccination campaigns as a response to the outbreak, due to insecurity issues the vaccination campaigns were implemented only in the southern governorates but not in northern governorates. As a result, no more, cases were detected in the southern governorates while the majority of confirmed cases were detected in the northern governorates.

Use of mOPV2 in HOA countries and high population movement across Yemen was the source of SL2 that diverged and circulated in the country in children with low immunity against this type, so changing to nOPV2, which is more genetically stable, in response campaigns will decrease this risk [19, 25, 26]. 

The cVDPV2 was isolated from environmental samples after an average of 7 months after sample collection. Environmental surveillance is an effective tool in polio eradication for early detection and monitoring during and after the outbreak, but the specimen’s transportation is still a big challenge in Yemen.

### Recommendations

Maintaining a high level of immunization coverage is essential to preventing the emergence and circulation of VDPVs. Achieving high levels of routine vaccination coverage with second doses of IPV all over the country. Environmental surveillance samples transportation should be radically resolved, and the system should be expanded in such a risky country. Timely and large-scale immunization campaigns should be implemented in response to cVDPV2 outbreaks, with the use of nOPV2 instead of tOPV and mOPV2 in SIAs to decrease the risk of the new emergence of VDPV2.

Advocacy, community leadership mobilization and fighting rumors against immunization should be utilized, especially in the northern part of the country.

### Electronic supplementary material

Below is the link to the electronic supplementary material.

Supplementary Material 1

Supplementary Material 2

## Data Availability

All relevant data are presented in this paper, and more information can be provided upon reasonable request from the corresponding author.

## Abbreviations

AFP: Acute flaccid paralysis
aVDPV: Ambiguous vaccine-derived poliovirus
bOPV: Bivalent oral polio vaccine
BMFS: Bag Mediated Filtration System
CDC: Centers for Disease Control and Prevention
cVDPV: Circulating vaccine-derived poliovirus
ES: Environmental Sample
ITD: Intrataypic differentiation
iVDPV: Immunodeficiency-related vaccine-derived poliovirus
IPV: Inactivated Polio Vaccine
GPEI: Global Polio Eradication Initiative
HOA: Horn of Africa
mOPV2: Monovalent Oral Polio Vaccine type 2
NPEC: National Polio Expert Committee
nOPV2: Novel Oral Polio Vaccine type 2
Nt: nucleotides
ONPL: Oman’s National Polio Lab
OPV: Oral polio vaccine
RI: Routine immunization
SIAs: Supplementary activities
SL1&SL3: Sabin like 1 and 3
SOPs: Standard Operational Procedures
tOPV: Trivalent oral polio vaccine
VDPV1,2: Vaccine-derived poliovirus type 1, 2
WHO: World Health Organization
