# Supplementary material for: Circulating vaccine derived polio virus type 2 outbreak and response in Yemen, 2021–2022, a retrospective descriptive analysis
Source: BMC Infect Dis. 2024 Mar 15;24:321. doi: 10.1186/s12879-024-09215-1 (PMC10943856; doi:10.1186/s12879-024-09215-1)
Supplement: Supplementary file 1 — Supplementary Material 1 [file 12879_2024_9215_MOESM1_ESM.docx]

| Supplementary Table 1: Distribution of cVDPV2 cases by governorate August 2021to December 2022. | | | | | | | | | | | | | | | | | | | | |
| --- | --- | --- | --- | --- | --- | --- | --- | --- | --- | --- | --- | --- | --- | --- | --- | --- | --- | --- | --- | --- |
| Years | 2021 | | | | | Total 2021 | 2022 | | | | | | | | | | | Total 2022 | | overall |
| Governorate | AUG | SEP | OCT | NOV | DEC |  | JAN | FEB | MAR | APR | MAY | JUN | JUL | AUG | SEP | OCT | DEC |  |  |  |
| **SOUTH** |  |  |  |  |  |  |  |  |  |  |  |  |  |  |  |  |  |  | |  |
| Taiz | 1 | 1 |  |  |  | 2 | 1 |  |  |  |  |  |  |  | 1 |  |  | 2 | | 4 |
| Marib |  | 1 |  | 4 | 2 | 7 |  |  |  |  |  |  |  |  |  |  |  | 0 | | 7 |
| Abyan |  | 1 | 1 |  | 3 | 5 |  | 1 |  |  |  |  |  |  |  |  |  | 1 | | 6 |
| Aden |  | 1 |  | 1 |  | 2 |  |  |  |  |  |  |  |  |  |  |  | 0 | | 2 |
| Al Dhale |  |  | 1 | 1 |  | 2 |  |  |  |  |  |  |  |  |  |  |  | 0 | | 2 |
| Hadramoat Say'un |  |  |  |  | 1 | 1 |  |  |  |  |  |  |  |  |  |  |  | 0 | | 1 |
| Lahj |  |  | 1 | 2 | 3 | 6 | 2 | 1 |  |  |  |  |  |  |  |  |  | 3 | | 9 |
| Shabwah |  |  |  |  |  | 0 | 1 |  |  |  |  |  |  |  |  |  |  | 1 | | 1 |
| **NOURTH** |  |  |  |  |  |  |  |  |  |  |  |  |  |  |  |  |  |  | |  |
| *Taiz |  |  | 3 |  | 1 | 4 |  |  |  |  |  |  |  |  |  |  |  | 0 | | 4 |
| *Marib |  |  |  |  |  | 0 |  | 1 |  |  |  |  |  |  |  |  |  | 1 | | 1 |
| Al Baida |  |  |  |  | 1 | 1 | 4 | 1 | 3 | 3 | 3 | 1 |  |  |  |  |  | 15 | | 16 |
| Al Hodaidah |  |  | 3 | 2 | 6 | 11 | 5 | 2 |  |  |  |  |  | 1 |  |  | 1 | 9 | | 20 |
| Al Jawf |  |  | 1 | 2 | 5 | 8 | 4 |  |  |  |  |  |  |  |  |  |  | 4 | | 12 |
| Al Mahwait |  |  |  |  |  | 0 | 1 | 1 |  |  |  | 2 | 1 | 2 | 1 |  |  | 8 | | 8 |
| Amaran |  |  |  | 2 | 2 | 4 | 1 | 1 | 3 | 1 | 2 | 1 |  |  |  |  |  | 9 | | 13 |
| Dhamar |  |  | 1 | 1 |  | 2 | 3 | 6 | 11 | 3 | 1 |  |  | 4 | 1 |  |  | 29 | | 31 |
| Hajjah |  |  |  |  |  | 0 | 3 | 1 | 1 |  | 1 | 1 |  | 2 | 1 | 2 |  | 12 | | 12 |
| Ibb |  |  |  |  |  | 0 | 2 | 1 | 2 | 4 | 3 | 4 | 2 | 1 |  |  |  | 19 | | 19 |
| Sa'adah |  |  | 3 | 3 | 4 | 10 | 5 | 1 |  |  |  | 1 |  |  |  |  |  | 7 | | 17 |
| Sana'a |  |  |  |  |  | 0 | 1 | 3 | 3 | 4 | 3 | 4 | 5 | 1 |  | 1 |  | 25 | | 25 |
| Sana'a city |  |  |  |  |  | 0 | 2 | 1 | 4 | 2 | 1 |  | 4 | 2 |  |  | 1 | 17 | | 17 |
| Grand Total | 1 | 4 | 14 | 18 | 28 | 65 | 35 | 21 | 27 | 17 | 14 | 14 | 12 | 13 | 4 | 3 | 2 | 162 | | 227 |
| *Taiz and Marib governorates have districts in both north and south | | | | | | | | | | | | |  |  |  |  |  | |  |  |
