# Supplementary material for: Circulating vaccine derived polio virus type 2 outbreak and response in Yemen, 2021–2022, a retrospective descriptive analysis
Source: BMC Infect Dis. 2024 Mar 15;24:321. doi: 10.1186/s12879-024-09215-1 (PMC10943856; doi:10.1186/s12879-024-09215-1)
Supplement: Supplementary file 2 — Supplementary Material 2 [file 12879_2024_9215_MOESM2_ESM.docx]

| Supplementary Table 2: Environmental samples, Date of collection, shipments and average of time delay from sample collection to receiving lab results , Yemen 2021-2022 | | | | | | | | |
| --- | --- | --- | --- | --- | --- | --- | --- | --- |
| Sample Collection | | Sample Shipment | | Average of Time delay between steps | | | Overall | |
|  |  |  |  |  |  |  | SColl – LR | |
| Year | Month | No. | Date | SCol.- SShip. | SShip.-SLabR. | SLabR.- LR | Days | Months |
| 2021 | Jul | 2 | 12-09-21 | 67 | 12 | 104 | 183 | 6 |
|  | Aug | 3 | 18-11-21 | 84 | 72 | 96 | 252 | 8 |
|  | Sep | 2 |  | 53 | 72 | 96 | 221 | 7 |
|  | Oct | 2 |  | 88 | 41 | 90 | 218 | 7 |
|  | Nov | 3 | 22-03-22 | 114 | 9 | 83 | 206 | 7 |
|  | Dec | 3 |  | 85 | 9 | 141 | 235 | 8 |
|  | Total | 15 |  | 84 | 35 | 103 | 221 | 7 |
| 2022 | Jan | 3 | 22-03-22 | 53 | 9 | 141 | 203 | 7 |
|  | Feb | 3 |  | 150 | 8 | 144 | 302 | 10 |
|  | Mar | 3 |  | 123 | 8 | 144 | 275 | 9 |
|  | Apr | 3 |  | 93 | 8 | 144 | 245 | 8 |
|  | May | 1 |  |  |  |  |  |  |
|  |  | 2 | 18-12-22 | 271 | 16 | 64 | 351 | 12 |
|  | Jun | 3 |  | 263 | 27 | 19 | 308 | 10 |
|  | Jul | 2 |  | 147 | 33 | 13 | 193 | 6 |
|  | Aug | 3 |  | 114 | 33 | 13 | 160 | 5 |
|  | Sep | 3 |  | 82 | 33 | 13 | 128 | 4 |
|  | Oct | 3 |  | 53 | 33 | 13 | 99 | 3 |
|  | Nov | 3 | 06-06-23 | 21 | 33 | 13 | 67 | 2 |
|  | Dec | 1 |  | 171 | 20 | 24 | 215 | 7 |
|  | Total | 33 |  | 121 | 21 | 67 | 210 | 7 |
| Grand Total | | 48 |  | 109 | 26 | 79 | 213 | 7 |
| SColl. = sample collection, SShip. = sample shipment, SLabR. = sample lab received, LR = Lab result | | | | | | | | |
